# Johne’s disease in Irish dairy herds: considerations for an effective national control programme

**DOI:** 10.1186/s13620-020-00166-y

**Published:** 2020-08-14

**Authors:** A. G. Jordan, L. R. Citer, C. G. McAloon, D. A. Graham, E. S. G. Sergeant, S. J. More

**Affiliations:** 1 c/o Ausvet Pty Ltd, Canberra, Australia; 2 Animal Health Ireland, 2-5 The Archways, Carrick on Shannon, Co. Leitrim, N41 WN27 Ireland; 3 UCD School of Veterinary Medicine, University College Dublin, Belfield, Dublin, D04 W6F6 Ireland; 4 UCD Centre for Veterinary Epidemiology and Risk Analysis, UCD School of Veterinary Medicine, University College Dublin, Belfield, Dublin, D04 W6F6 Ireland

**Keywords:** Johne’s disease, control programme, Ireland, dairy industry

## Abstract

The Irish dairy industry has established a reputation for the production of safe and healthy dairy products and is seeking to further expand its export market for high value dairy products. To support its reputation, stakeholders aim to control Johne’s disease. To assist decision-makers determine the most appropriate design for an Irish programme, a narrative review of the scientific literature on the epidemiology of Johne’s disease, and selected control programmes throughout the world was undertaken. Two modelling studies specifically commissioned by Animal Health Ireland to assess testing methods used to demonstrate confidence of freedom in herds and to evaluate a range of possible surveillance strategies provided additional information. The majority of control programmes tend to be voluntary, because of the unique epidemiology of Johne’s disease and limited support for traditional regulatory approaches. While acknowledging that test performance and sub-clinical sero-negative shedders contributes to the spread of infection, a range of socio-political issues also exist that influence programme activities. The paper provides a rationale for the inclusion of a Veterinary Risk Assessment and Management Plan (VRAMP), including voluntary whole herd testing to identify infected herds and to support assurance-based trading through repeated rounds of negative testing, national surveillance for herd-level case-detection, and improved understanding of biosecurity management practices. Identification and promotion of drivers for industry and producer engagement in Ireland is likely to guide the future evolution of the Irish Johne’s Control Programme (IJCP) and further enhance its success. The provision of training, education and extension activities may encourage farmers to adopt relevant farm management practices and help them recognize that they are ultimately responsible for their herd’s health and biosecurity.

## Background

The dairy industry in the Republic of Ireland is differentiated by its production system from the dairy industries in many countries within the European Union and beyond. The industry is based on an extensive pasture-based grazing system for most of the year with cows being housed for a limited period, typically 6–8 weeks during winter. Calving commences during this time and continues into early spring. Most calves are born in a short and intensive calving period of six to eight weeks from January to March.

As an exporter of dairy products, the Irish industry has established a reputation for food safety. Utilising a sustainable production system, the industry is seeking to continue to grow and extend its market share in high value products. It has been actively encouraged to do so by government policies [1–4].

Following a long history of limited growth in production due to the operation of a European Union quota system (abolished in 2015), the dairy industry has undergone significant expansion in recent years, growing the volume of milk produced from 6.3bn litres in 2015 to 8bn litres in 2019. Since 2010 the economic value of dairy exports has grown by 78%, 11% of this growth occurring in 2019 alone, to greater than €4 billion in value [5, 6], further increasing its significance to the agri-food sector in Ireland.

With respect to dairy products, the United Kingdom (UK) remains the largest single trading partner of Ireland, followed by China, continental Europe and the United States of America. The current changing international trading environment caused by the decision of the UK to leave the EU along with rising international protectionism has the capability of affecting existing export market relationships. However, any loss of access to individual markets may be countered by world population growth which is likely to create growth in potential new markets. These new markets may bring with them a requirement for additional product specifications. Ireland has an interest in capitalising on its reputation as a producer of safe, environmentally sustainable dairy products in any negotiations with emerging trading partners and national animal health programmes serve to support such claims.

One of the challenges of developing a national Johne’s disease control programme for the Irish dairy industry is to ensure that it appropriately reflects the social and political priorities in which it will operate. To ensure any programme is fit-for-purpose, factors as diverse as an industry’s structure, underlying national disease prevalence, long-term government policies, and market access all influence the decision-making process.

The aim of this paper is to present considerations for an effective national control programme for Johne’s disease in Ireland. These related to agreed programme objectives, the scientific basis for the control of Johne’s disease, regional and national control programmes in other countries and known challenges and constraints. Recommendations for a credible and sustainable control programme for the Irish dairy industry are presented, which were subsequently adopted by Irish stakeholders as the basis for the national Irish Johne’s Control Programme (IJCP).

## The approach

A narrative review of the scientific literature was undertaken to:

- Review the current scientific basis for the control of Johne’s disease in developed countries with long-standing control programmes, which is presented in Appendix A.
- Identify any additional large-scale control programmes internationally since the previous review undertaken by Animal Health Ireland (AHI) [7], and the elements that comprise these programmes. More recent information about these programmes was obtained from an internal paper developed by AHI in 2017 and from the scientific literature [8].

The biennial colloquium of the International Association for Paratuberculosis (IAP), a not-for-profit scientific association ‘devoted to the advancement of knowledge and scientific achievement toward the eradication of paratuberculosis in domestic livestock’, includes a stream on control programmes or aspects of Johne’s disease control. A review of conference proceedings determined that relatively few countries have embarked on large-scale control programmes, and of those even fewer have attempted to determine the long-term efficacy of control measures. Despite the interest in MAP infection as a potential public health issue, Whittington et al. confirmed that out of 48 countries surveyed, 22 indicated the presence of some form of control for MAP, and of these 13 have undertaken an evaluation [8].

The review by Geraghty et al. (2014) [7] provides a comprehensive overview of control programmes operating at the time, and was used to inform initial discussions among Irish stakeholders within the AHI Johne’s disease Implementation Group (JDIG) leading to the formation of programme objectives for a pilot programme. Once these objectives had been agreed, the narrative review of control programmes was adjusted to consider programmes with similar objectives, with particular consideration of control options, and of challenges and constraints to programme credibility and sustainability. Further, two modelling studies were specifically commissioned by AHI to assess testing methods used to demonstrate confidence of freedom in herds [9] and to evaluate a range of national surveillance strategies for the detection of infected herds [10]. Collectively, this information was used to inform recommendations to the JDIG on the design and structure of a credible and sustainable Irish Johne’s disease control programme.

## Agreed programme objectives

The Irish Department of Agriculture, Food and the Marine (DAFM) has adopted a partnership approach with the dairy industry in the management of non-regulated animal health issues. Established in 2009, AHI is a stakeholder organisation with a remit to engage interested parties through a consensus-based approach to decision making and in consultation, determine the objectives, structure and scope of animal health programmes which it coordinates [11]. This includes the development and implementation of animal health programmes for non-regulated animal health conditions [7, 12].

In accordance with its remit, AHI conducted an industry-wide stakeholder consultation to identify dairy industry animal health priorities in 2009. An expert Delphi approach was conducted with animal health experts based in Ireland. Three rounds of consultation were carried out before a consensus was reached. At the same time, prioritisation surveys were also conducted with Irish farmers [12]. Johne’s disease was ranked highly despite the relatively low prevalence of disease nationally [12]. However, stakeholders recognised that the situation could potentially deteriorate and in order to re-assure trading partners and stakeholders, a long-term programme to control the spread of Johne’s disease within the dairy industry was required. It was further proposed that such a programme should limit the spread of infection within the national dairy herd and in doing so provide assurance to consumers that Irish dairy products maintained a high standard of food safety.

From the outset, stakeholders accepted the importance of an ‘evidence-based’ approach to Johne’s disease control which would, over time, reduce the prevalence of disease nationally and demonstrate an increasing confidence (assurance) that products, including animals, derived from Irish herds were free of infection with *Mycobacterium avium* subspecies *paratuberculosis* (MAP). Stakeholders also recognised the importance of having a national programme which provided pathways for both test-negative and test-positive herds with the aim of protecting herds from the risk of introducing infection (bioexclusion) as well as reducing the incidence of infection (biocontainment) in herds that had tested positive for the disease.

In developing an appropriate approach for Ireland, initially as a pilot programme which was in place from November 2013 to December 2016, stakeholders identified the importance of reviewing existing international approaches and consciously adopting or adapting these approaches to the distinctive requirements of Irish stakeholders and this was published in 2014 [7]. At the conclusion of the pilot Johne’s disease control programme, this body of knowledge was updated in 2017 by AHI (unpublished internal paper) and expanded in 2019 by Whittington et al. [8].

On the basis of these updates and the learning from the pilot programme, stakeholders reaffirmed the objectives for a future voluntary national control programme which could enable the aims which had been identified by stakeholders, viz.:

1. to identify test-negative herds and provide farmers with the tools and knowledge to increase their confidence of freedom over time;
2. to identify infected herds and provide farmers with the tools and knowledge to control the disease and reduce within-herd transmission;
3. to further underpin the quality of Irish dairy and beef produce in the international marketplace; and
4. to improve calf health and farm biosecurity in participating farms.

## Regional and national Johne's disease control

Despite the challenges of incomplete epidemiological knowledge and the reliance on imperfect tests, concern over the economic impacts and potential public health implications of MAP infection has been sufficient to lead to the development of national or regional programmes for Johne’s disease control in many countries [8].

Control programmes throughout the world are largely voluntary and mostly involve risk categorisation or classification, with recommendations made on the basis of test results and a Veterinary Risk Assessment and Management Plan (VRAMP) or similar [7, 13]. Utilising the available epidemiological knowledge, control programmes most often incorporate the elements of herd-level risk assessments, on-farm biocontainment and the promotion of herd assurance, by way of a variety of qualitative and quantitative approaches, which usually include regular whole-herd testing.

Serum or milk serology is mostly used for testing for herd classification, because of its convenience and low cost.

### Commonly identified elements of Johne’s control programmes

#### Veterinary Risk Assessment and Management Plans

VRAMPs are a tool to evaluate individual farm risk for the introduction and spread of MAP and educate producers on steps they can take to reduce these risks. They are typically conducted by trained private veterinary practitioners and involve an on-farm questionnaire on the management practices pertinent to MAP control. The responses to the questionnaire enable private veterinary practitioners to determine the sources of risk for the entry and spread of MAP in a herd. Farm-specific recommendations for MAP prevention and control are developed in conjunction with the farmer.

A VRAMP can be an effective tool provided the survey questions align well with established risk factors and producers follow the recommendations [14]. In Ontario, Canada participating farms in a voluntary programme based around risk assessment reduced their MAP-antibody milk ELISA-positive prevalence and improved risk assessment scores, which were correlated to improved management practices [15]. Regular review of individual farm VRAMPs enables private veterinary practitioners to assess farmer uptake of recommendations and allows further changes to management plans to be made if required. For consistency, consecutive VRAMPs should be performed by the same private veterinary practitioners where possible [16]. Evaluation of the effectiveness of VRAMPs (reduction in farm prevalence or improved herd management) requires sufficient passage of time for a large proportion of heifers born under new management practices to be present in the herd(s) [17].

#### Assurance testing

Herd owners can reduce the likelihood of MAP being introduced by implementing bio-exclusion practices, such as closing the herd or restricting introduction of animals to only those from low-risk herds. This requires identification of a pool of low-risk herds as a source of replacement animals and is a key role for test-based assurance programmes. Imperfect test characteristics and sampling strategies mean that definitive proof of herd freedom is seldom achievable. Instead, testing is carried out to demonstrate confidence that infection is not present above a specified minimum prevalence (design prevalence) [18]. This is commonly referred to as ‘proof of freedom’ testing. The degree of confidence in freedom is dependent on factors including the sampling strategy and interval employed, the expected true prevalence of the disease in the population sampled and diagnostic test performance (sensitivity and specificity) [19, 20]. In the context of MAP, confidence of freedom is also greatly influenced by the purchasing behaviour of the herdowner prior to and in the intervening period between testing [9, 21].

Poor sensitivity of currently available MAP tests may result in many false negatives, which increases the number of rounds of testing required (and therefore cost) for a test-negative herd to acquire a reasonable confidence of freedom. Imperfect test specificity is also problematic and follow-up confirmatory testing of positive test results adds to the cost and complicates herd assurance determination. Despite these limitations, moderate increases in confidence of freedom can be achieved over several years of repeated herd-testing [9, 21].

In countries where classification is undertaken, herds may be classified according to prevalence level or by the number of rounds of negative testing, so that the levels of risk they present are differentiated qualitatively. This permits the use of defined assurance pathways, applicable to all herd owners and in which producers can progress from higher risk to lower risk categories/scores (for example, progressing from high test-positive prevalence to low prevalence and then to test-negative). However, systems of categorisation which are based on testing only overlook the role of livestock introductions as a source of infection risk.

Using data from the Irish pilot programme (2013–2016), a stochastic simulation model was developed to examine the impact of alternative testing strategies on confidence of herd freedom, accounting for the risk attributable to stock introductions from the general population of herds. This work found that a single annual herd ELISA test (milk or serum) of cattle ≥2 or ≥ 3 years could achieve adequate confidence within a reasonable timeframe [9]. This scenario provides a more practical strategy than that used in the pilot programme in Ireland, where biannual milk testing was used. To reduce costs, a further reduction in test frequency, decreased sample size or alternative herd-based tests could be considered for maintenance of confidence once a specified value has been met.

#### National surveillance testing (case-detection)

Case-detection is undertaken to identify infected herds for further action. In a voluntary control programme, however, surveillance is typically limited to participating herds. Therefore, a national surveillance project provides the opportunity for more comprehensive coverage for case-detection, since all herds or a larger portion of the national herd are tested, rather than only the participating herds.

Many methods exist for case-detection, including whole herd milk or serum serology (of individual cattle), bulk tank milk serology, individual or pooled faecal culture/polymerase chain reaction (PCR), and environmental culture/PCR [7, 8].

Sergeant et al. (2019), modelled the above surveillance strategies and found that none are ideal for widespread application as part of a national surveillance programme for detecting MAP-infected herds in Ireland [10]. Herd testing with individual milk and/or serum ELISA or pooled faecal testing were the most effective methods for detection of MAP-infected herds, although they are costly for widespread application.

Environmental sampling showed promise as an alternative for national case-detection if samples could be pooled for testing but is otherwise relatively expensive and requires further validation and determination of appropriate pooling rates under Irish conditions.

Bulk milk tank testing is the lowest cost option and may be useful, primarily for detection of higher-prevalence herds to allow targeting of advice to farmers to reduce within-herd prevalence and prevent spread to other herds, provided a method for resolving false positive tests could be identified. Abattoir sampling of cull cows using a serum ELISA test was a lower-cost option but had a lower herd-level sensitivity and very poor positive predictive value compared to other surveillance methods. This would result in many undetected false-negative herds and up to 50% false-positive herds in the absence of the possibility of performing confirmatory testing, requiring follow-up testing of positive herds to clarify infection status.

### Regulatory control of Johne’s disease

Individual countries vary in their approach to the regulatory control of MAP. The World Organisation for Animal Health (OIE) Terrestrial Animal Health Code Chapter on Paratuberculosis provides little guidance on movement recommendations for Johne’s disease and this has led to some importing countries applying specific requirements for livestock entering the country. In the absence of a formal recommendation from the OIE, the IAP published *Guidelines for certification with respect to the movement of livestock for Mycobacterium avium subsp. paratuberculosis (MAP) infection* [22]. Some countries with a significant export of live animals to certain destinations require notification of infected animals to veterinary authorities, usually where there is a requirement to certify products or animals for export. However, unless there is an export trading requirement, regulatory components of control programmes may be considered overly burdensome by producers and lead to reduced participation in MAP control [23].

## Challenges and constraints

### Challenges to implementation

Poor reliability of tests, particularly for animals with early-stage infection, a lack of producer engagement and support, and inadequate funding appear to have been the most common constraints identified by the coordinators of control programmes internationally. In general, progress towards control and in particular, the lessons learned from national programmes have not been widely reported, so that the long-term efficacy of control programmes is largely unknown [8, 24].

The level of farmer engagement and participation in a voluntary control programme is dependent on the strength of drivers (cultural and financial) and in the perceived benefits to the individual farmer and the industry as a whole. In some countries, notably the Netherlands, where the dairy industry is the major cattle industry, the participation rate is very high because participation in the programme with exclusion of milk from test-positive animals is a condition of supply. In an environment where one processor dominates the market, this provides a strong driver for farmers [7].

In some countries, financial assistance from government or industry bodies is provided to farmers for participation. However, more recently there has been a global trend towards industry or producer-funded coordination of Johne’s disease control programmes. Where government funding is made available, it generally complements funding provided by industry bodies or requires individuals to cover a portion of their own costs. Funding from government is usually directed towards research, and the maintenance of testing protocols and standards as well as some administrative support. Costs for programme activities, such as testing or VRAMPs, are largely borne by farmers.

### Drivers for producer engagement in control programmes

Since many national programmes are voluntary, they rely on farmer engagement, participation and uptake of preventative recommendations for progress towards infection control nationally. Farmer behaviour in relation to these matters has become an important area of research. Although financial considerations are important for some farmers, personal values and belief systems also influence their behaviour [25]. The perceived level of disease in a country and a farmer’s experience of controlling other diseases impacts farmers’ views about the importance of control programmes, and thus the likelihood of success [26].

Several psychological frameworks are available for evaluating farmer attitudes, such as the theory of planned behaviour [27, 28].

Even if farmers regard recommendations as reasonable and feasible, they may not implement them [14]. Some producers may not see Johne’s disease as worth addressing unless the prevalence of clinical disease on-farm is high, necessitating excessive culling [29].

Familiarity of farm managers with Johne’s disease or prior diagnosis did not significantly influence the use of preventative management strategies in the United States of America [30]. In Alberta, Canada, larger herd sizes, higher self-assessed knowledge, greater knowledge of the provincial control programme and a better relationship with their private veterinary practitioners were associated with programme participation. Participants were not more concerned with the effects of Johne’s disease on the dairy industry than non-participants. Reasons for a lack of participation were the required time commitment and adoption of a ‘wait and see’ approach to evaluate if the programme worked for other farms [31]. A Canadian experiential learning programme (Farm Focus) for farmers that included exemplar farm tours, group work/discussions, meetings with experts and planning sessions reported success in improving knowledge levels and MAP management practices [32].

Government compensation or other financial assistance schemes to improve farmer willingness to engage in MAP control efforts has been used from time to time, but the outcome of such schemes has not been widely reported. When provided, financial assistance can be aimed at recovering the cost of the VRAMP, testing or to assist in implementing control actions, such as culling.

### Lessons from established control programmes

In a survey of 48 countries, 22 were found to have a programme for the control of paratuberculosis in operation at either regional or national level. There was an association between the presence of a control programme and presence of advanced veterinary services in the country [8]. The herd level prevalence of Johne’s disease varied between countries, and a quantitative measure was not readily available for many countries. However, details of some national programmes have been published and evaluated including the Dutch, Danish and Australian programmes.

The Dutch Milk Quality Assurance Programme is an example of a programme operating where infection is endemic. There is a high level of participation and associated reduction in herd-level prevalence in some herds due to market expectations and the requirement from the principal milk processors for supplier participation. Herds must be test-negative or have culled test-positive animals before milk will be collected from the farm. Initially milk processors funded most costs for producer participation in the Milk Quality Assurance Programme [33], which increased the participation rate dramatically. After some time, processors reduced financial input to cover only the participation fee. Herd-level apparent prevalence decreased over a ten-year period in dairy herds that entered the programme [34].

The Danish Programme also operates in a high prevalence environment and has made considerable advancement in the control of Johne’s disease through quarterly milk testing and the classification of animals based on their test results. Colour coding is used to identify risk levels and cows in the highest risk group are culled. Over time the programme administrators have noted a decline in participation because farmers do not see the benefit of control in the absence of clinical disease.

The partially regulated Australian programme which operated in a low prevalence environment was not sustainable in the longer term because: 1) the perceived benefits, particularly to the predominantly beef industry, were limited compared to the regulatory restrictions, and 2) the cost of participation and potential consequences of detecting MAP (e.g. quarantine and subsequent restrictions on animal movements) in participating herds. This led to a lack of producer support [23]. During the time of operation of the beef programme, the dairy industry progressively moved towards deregulation by implementing a qualitative risk assurance programme which continues to operate with limited uptake. Both of these programmes have now been subsumed into a national biosecurity programme with specific trading requirements for interstate movement of livestock.

## Designing a credible and sustainable control programme for the Irish dairy industry

After reviewing the literature and considering the current scientific basis for Johne’s control, the modelling work undertaken on behalf of the Irish industry and the programme objectives identified by stakeholders, a number of programme activities were identified as common to programmes which had achieved some level of control:

1. The use of VRAMPs to provide a platform for veterinary guidance on improving individual farm biosecurity and preventing MAP introduction (bioexclusion). Regular VRAMPs have been shown to improve farmer-initiated biosecurity, both bioexclusion and biocontainment. A concurrent communication programme to engage farmers in actively implementing any changes to modify current farm management practices is required since the success of a voluntary programme is highly dependent on farmer participation and engagement. To remain effective, VRAMPs require regular review to determine farmer uptake of recommendations, correct any omissions and to capture changes in farm circumstances or management systems and identify any emerging risks.
2. The regular use of herd screening tests based on individual animal tests for the purpose of determining herd assurance and the early detection of infection.
3. The establishment of an objective quantitative measure of herd assurance.
4. A national surveillance project for herd-level case-detection, in association with voluntary action by producers. Diagnostic test performance and the cost of testing may limit the applicability of widespread testing for national surveillance, however the use of low-cost methods (e.g. bulk milk testing or environmental testing) to detect high-prevalence (high-risk) herds may enhance a national programme by identifying herds that have yet to engage in Johne’s control, for targeted action.

Vaccination is not considered within this paper because of potential interference with test performance for bovine tuberculosis (bTB), noting national efforts towards bTB eradication. Regulated MAP control is also not being considered in Ireland.

### Alignment to programme objectives

The future progress of a national Johne’s disease control programme cannot be predicted reliably without complex modelling. Nonetheless, it is likely that a combination of VRAMPs (with a focus on biosecurity and calf hygiene to mitigate risk), herd assurance based on livestock introductions and herd-level testing, and national surveillance for herd-level case-detection is most likely to meet the stated programme objectives. Recommendations for programme activities to robustly address each of these objectives are considered here and presented in Table 1.

**Table 1:** Programme option alignment with Irish Johne’s Control Programme objectives

Programme objectives	VRAMP alone	Voluntary test-based assurance programme with VRAMP	National surveillance for case-detection and voluntary farmer action	Combination of VRAMP, voluntary assurance and national surveillance
1. Enhance the ability of participating farmers to keep their herds clear of MAP infection	*Partially*. Bioexclusion recommendations are provided, but farmers are unaware of their herd infection status or the risk from source herds for introductions in the absence of assurance testing.	*Yes*. Low-risk herds participating in programme can be identified as a source of new stock if required. VRAMP bioexclusion recommendations complement this. Should be supported by a farmer declaration of herd assurance score.	*No*. Farmers do not have information on other herds’ status, unless case-herd farmers volunteer this information. Engagement with the programme after positive results is not mandatory. The test sensitivity of some case detection methods for example Bulk Tank Milk testing precludes identifying all case herds.	*Yes*, largely through voluntary assurance programme and VRAMP.
2. Enable participating farmers to reduce the level of infection in their herds, where present	*Partially*. Biocontainment recommendations are provided, but farmers are unaware of their herd infection status in the absence of testing.	*Yes*. Participating herds that are identified as infected can implement control measures and within-herd prevalence may be determined. VRAMP biocontainment recommendations complement this.	*Partially*. Infected herds can be identified, and farmers contacted for general education on control measures. Uptake of control recommendations is dependent on the farmer and within-farm prevalence and specific farm context and management practices are likely unknown.	*Yes*, identification of infected herds by voluntary assurance testing and VRAMP to provide guidance on control measures, supplemented by national surveillance for case detection
3. Provide additional assurance to the marketplace (domestic and international) in relation to Ireland’s efforts to control MAP infection	*No*. Limited value without assurance testing or national surveillance.	*Partially*. Milk processors can source milk from herds identified as low risk for sensitive markets. Provides some case-detection of infected herds, but unless participation rates are high does not provide broad case-detection or national prevalence estimates to assure trading partners.	*Partially*. More comprehensive case detection may be achieved than through a voluntary assurance programme. High-prevalence (high-risk) herds could be detected for voluntary action and exclusion from sensitive markets (e.g. through bulk milk testing surveillance)	*Yes*, depending on market requirements. Voluntary assurance programme can be enhanced by national surveillance to detect high prevalence (high-risk) herds to encourage participation in assurance programme and VRAMP. The combination of test-based voluntary assurance and national case-detection provides more information to share with trading partners/markets, if required.
4. Improve calf health and farm biosecurity in participating farms	*Yes*. Bioexclusion and biocontainment recommendations are given, however these may be general in nature, without knowing herd infection status.	*Yes*. VRAMPs address this objective, plus regular herd-testing to identify high-risk animals for culling.	*Partially*. General recommendations given for identified case-herds, but unlikely to consider within-herd prevalence and individual farm context and management practices.	*Yes*, through VRAMP, plus regular herd-testing to identify high-risk animals for culling.

#### Objective 1: Enhance the ability of participating farmers to keep their herds free from MAP infection

A voluntary test-based herd assurance programme consisting of varying levels of herd assurance (e.g. herd assurance scores) can meet this objective by identifying low-risk (high assurance) herds from which animals could be sourced as well as providing defined pathways to improve assurance levels over time for participating herds. To reduce the cost of on-going participation and encourage producers to progress towards higher herd assurance levels, a maintenance option is being explored based on alternative testing strategies (e.g. fewer animals, biennial testing or environmental testing).

A risk-based trading (assurance) system based on herd testing and a consideration of the number of animals introduced to the vendor’s herd over the preceding five-year period (underpinned by voluntary farmer declarations of assurance scores) would provide a mechanism for farmers to trade with greater confidence. Risk mitigation practices generally recommend the purchase of animals from farms with a similar or better MAP risk-profile (that is, an equivalent or better herd assurance score). The inclusion of VRAMP information on a voluntary farmer declaration would provide additional information useful in informing prospective purchasers about the level of risk associated with the herds from which animals could be sourced.

National surveillance for case-detection using protocols such as bulk tank milk testing, without other complementary activities is not well aligned to this objective, as it does not provide farmers with sufficient information about the herd history to prevent ongoing spread of MAP through introductions of infected animals.

#### Objective 2: Enable participating farmers to reduce the level of infection in their herds, where present

A voluntary test-based assurance programme will detect some case herds, but, depending on participation level, may not be as encompassing as other national surveillance options. To extend the reach of a voluntary programme, surveillance using bulk milk testing is being implemented as it can provide broad, low-cost screening to identify high prevalence herds and encourage the herdowners to engage with the programme [10].

Targeted actions may include farmer education and the provision of training for animal health advisers including private veterinary practitioners on the conduct of detailed epidemiological investigations. Farmers will need encouragement within a formal control programme for ongoing support to carry out a VRAMP with the aim of managing infection risk and reducing within-herd prevalence over time.

Veterinary recommendations may include measures to minimize within-herd transmission (biocontainment) and ways to reduce on-farm prevalence. For herds where infection has been confirmed following a positive Bulk Tank Milk test, the advisory actions should focus on biocontainment and whole herd testing including the early removal of high-risk animals. These measures can reduce consequential risk to other herds through movement of stock or manure. VRAMPs should be regularly reviewed to encourage farmers to implement biocontainment recommendations.

Efforts to reduce infection levels in individual farms could be supported by voluntary periodic herd-level monitoring via environmental testing or whole herd-testing. Individual animal testing of high-risk animals or cohorts of animals in herds known to be infected may also be useful to confirm which animals are infectious and should be culled - an important component of on-farm control.

Implementation of a VRAMP in the absence of whole herd testing may have some effect on within-herd prevalence. However, whole herd testing continues to have an important role in the identification of infected and infectious animals and provides a useful method to monitor the effectiveness of management practices through a reduction in within-herd prevalence and the age at which animals are found to be test positive.

#### Objective 3: Provide additional assurance to the marketplace (domestic and international) in relation to Ireland’s efforts to control MAP infection

A combination of test-based herd assurance, herd-level case detection, action in infected herds and use of VRAMPs can provide additional assurance to the domestic and international marketplace. In the longer term, herd assurance programmes will provide milk processors with the ability to manage market risks. Herd-level case detection (national surveillance) may also be used nationally to monitor changes in herd-level prevalence over time, and measure progress towards programme objectives. Collectively these activities provide stakeholders and trading partners with greater confidence that high-risk (high prevalence) herds are being detected in a timely manner so that action can be taken to reduce the risk of MAP entering the milk production chain.

#### Objective 4: Improve calf health and farm biosecurity in participating farms

The provision of educational material or training as part of a broader communication strategy to promote biosecurity can address this objective. While general farmer education on MAP control and prevention strategies is useful in improving awareness, individual farm-specific recommendations are more appropriately provided through a VRAMP.

As the programme matures and continues to evolve, periodic monitoring of herd performance for specific parameters which have been identified as having an association with Johne’s disease, such as somatic cell count, calf morbidity and lameness, could complement VRAMPs and provide evidence of improvement in biosecurity and calf health [35].

In principle, improved calf hygiene measures implemented to control MAP may also lead to improved control of other calf pathogens. Improvements in calf health and farm biosecurity are also crucial components of Objectives 1 and 2 above.

## Conclusions

The above-mentioned programme recommendations were subsequently adopted by Irish stakeholders as the basis for the national IJCP, which commenced in 2018. Therefore, the IJCP includes four objectives which are being addressed through a combination of VRAMP, voluntary assurance and national surveillance. The programme is being coordinated within a cost-sharing model based on a government-industry partnership. In addition, the IJCP is underpinned by a comprehensive communication and social research programme to identify and then utilise the drivers and address the constraints which are identified to further engage farmer participation in Ireland. This is being complemented by high quality farmer education and training that takes into account these factors and the context in which farmers manage dairy herds in Ireland.

The development of quantitative and qualitative metrics to monitor and evaluate any MAP control programme is necessary to measure progress towards long-term aims, to ensure a programme is meeting the stated objectives and to identify aspects of the programme which may need modification over time. The control of Johne’s disease is a long-term proposition and it is anticipated that the IJCP will continually evolve to remain relevant and sustainable into the future.

## Figures and Tables

**Fig. 1:**
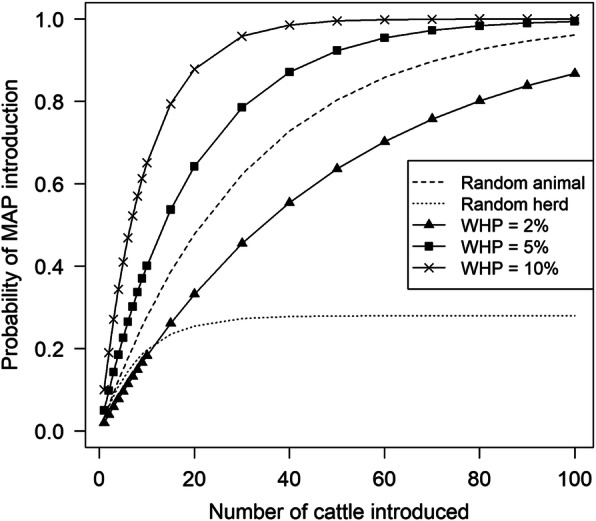
Probability of MAP introduction into an Irish dairy herd via new cattle introductions when herd-level prevalence = 0.28 and overall animal-level prevalence = 0.032 (prevalence estimates from McAloon et al. [37]). Random animal refers to introduction of an animal chosen at random from the national herd given the aforementioned prevalence values. Random herd refers to all introduced animals being from a single randomly chosen herd where herd-level prevalence = 0.28 and within-herd prevalence (WHP) = 0.114

**Fig. 2:**
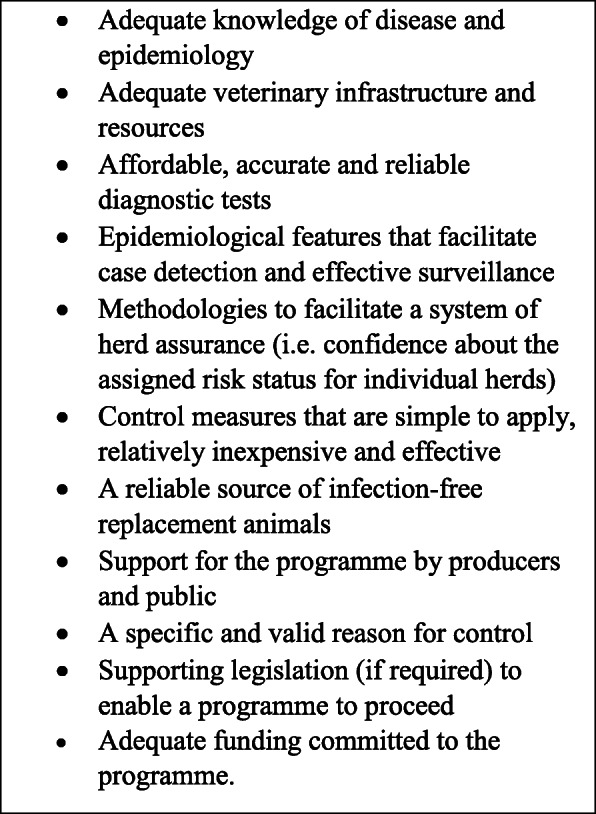
Factors that enhance the probability of a Johne’s disease control programme successfully controlling spread within and between herds

## Data Availability

Not applicable.

## Appendix

### Underpinning science relevant to Ireland

#### Epidemiology

Johne’s disease has a global distribution [24]. Prior to the mid-1990s, it was reported sporadically in Ireland. In 2005, dairy herd-level true prevalence was estimated at approximately 20.6% and animal-level true prevalence 2.9% [36]. Using Bayesian methods, McAloon et al. [37] estimated true prevalence for herds enrolled in the pilot Johne’s Control Programme during 2013–2014 to be 28% (95% posterior probability interval; 23, 34%) and animal-level prevalence to be 3.2% (95% posterior probability interval; 0.9, 14.5%) across all herds. However, it was noted that different statistical methods and populations were analysed for each of these estimates and that the findings should therefore be interpreted cautiously in terms of underlying trends.

#### Transmission within infected herds

MAP is shed in the faeces of infected cattle and transmitted via the faecal-oral route. The incubation period is long (typically 2–4 years) and cattle rarely show signs before two years of age [38]. Clinically affected cattle excrete larger numbers of MAP, but sub-clinically (latently) infected cattle are the greatest source of infections [38]. Young animals (< 6 months) are generally thought to be most susceptible [39]. A more recent experimental study showed that calves can become infected up to 12-months of age with both high and low MAP doses [40] and adults can become infected if exposed to large doses in highly contaminated environments [39]. Calves exposed to higher MAP doses early in life may shed more frequently than those exposed to lower doses [41]. MAP can remain viable in faeces for at least a year, particularly in shaded environments [42].

Suckling calves can be exposed to MAP via milk and colostrum or suckling contaminated teats. MAP is shed directly in milk or colostrum in a proportion of clinically or sub-clinically infected dams [43, 44]. Colostrum on commercial farms is frequently contaminated with faecal material [45] and a large proportion of MAP-positive colostrum is explained by environmental contamination from the retention of infectious adult animals [46]. There is an association between poor udder hygiene and MAP positivity in milk [47].

In-utero transmission can occur, and it is estimated that approximately 9% of foetuses from sub-clinically infected cows and 39% from clinically-infected cows are infected via this route [48]. Sheep strains of MAP cause disease in sheep and goats, but the risk of transmission to cattle is very low and only occurs sporadically [49]. Viable MAP has been retrieved from dust and aerosol transmission and intestinal infection via this route has been demonstrated experimentally [50]. The significance of this finding for field conditions is not yet known.

Spread of MAP between herds is primarily through the introduction of infected cattle [51]. Often these animals are sub-clinically infected and may have tested negative. MAP is also spread via transfer of colostrum or manure between farms (deliberate application or inadvertently through fomites) [52] and possibly through shared grazing.

#### Between herd transmission

Calf contact with adult cow faeces is the major risk factor for within-herd transmission on dairy farms [53, 54]. In Ireland, the use of calving pens for sick or lame cattle has also been identified as a herd-level risk factor [55].

The introduction of new cattle is the most well-established risk factor for between-herd transmission [51], although not identified in all studies [55]. Regular cattle introductions from herds of unknown MAP status increases the risk of a farm environment having culturable MAP [56]. Figure 1 illustrates the theoretical increase in probability of MAP introduction into an Irish dairy herd with an increase in number of cattle introductions. The probability of introduction is higher when cattle are introduced from a herd with a higher within-herd prevalence or from multiple different herds. A cross-sectional survey of 1004 US dairies found that a larger herd size, greater percentage of cows born at other dairies, group housing for peri-parturient cows and group housing for pre-weaned calves were positively associated with infected herds [30].

#### Diagnostic test performance

There are many individual animal tests for MAP which may also be used as group or herd level tests, including faecal culture, faecal PCR, serum antibody ELISA and milk antibody ELISA. Additional herd-level tests include environmental testing (culture or PCR) and bulk milk tank testing (antibody ELISA).

A knowledge of diagnostic test sensitivity and specificity and the factors that affect these parameters is critical in determining the design of control programmes including the selection of tests to be used [57]. For MAP, this choice has been complicated by the lack of a standardised evaluation protocol for tests for use in different populations [58]. Test performance varies between clinically affected, infected (but not necessarily infectious), infectious and non-infected cattle [58]. In general, available tests have imperfect specificity (with serological tests) and low diagnostic sensitivity, particularly for young, sub-clinically infected cattle. Sensitivity increases in older cattle [24] and those displaying clinical signs [59].

#### Control principles

The probability of successfully controlling spread within and between herds is considered to increase as more of the programme elements shown in Fig. 2 are implemented (adapted from Sergeant and Perkins, 2015 [57]).

National MAP control programmes have been implemented in many high-income countries with variable success. With respect to the factors in Fig. 2, they have been constrained by a lack of affordable, accurate and reliable diagnostic tests, challenges in obtaining or retaining support by farmers and other key stakeholders; relative complexity of control measures; and inadequate funding [24].

#### Knowledge gaps

The epidemiological challenges of MAP control are well recognised [24] and MAP has been well studied. Despite this, many knowledge gaps remain. It is not known whether elimination of MAP from a dairy herd is possible [60] and reports of successful attempts are not available in the scientific literature. Accurate estimates of MAP prevalence are required to assess the impact of control programmes over time [37]. In many countries, comparative prevalence estimates at different time points are not available and those that are often use a variety of diagnostic tests or methodologies.

The comparative importance of transmission routes is unclear. A recent Dutch study revealed that the risk of calves shedding MAP up to 2 years of age was not influenced by the presence of MAP in the colostrum or faeces of the dam in a contaminated environment [61]. Earlier studies reported that calves born to seropositive dams had 6.6 times the odds of being seropositive compared to those born to seronegative dams [62]. Patterson et al. (2019) noted a positive association between the dam’s ELISA test status at the time of birth, and also in a subsequent seroconversion [63].

Calf-to-calf transmission tends to be overlooked in MAP control programmes [64] and the risk of this pathway has not yet been quantified by longitudinal studies. The results of intrauterine infection on time of onset of shedding and progress of infection compared to infection soon after birth are also unknown.

More research is required to determine the significance of the role of dust in transmitting MAP via ingestion or inhalation under field conditions. The importance of different MAP genotypes, breed/genetic susceptibility and the role of wildlife in the spread of infection has not been well described [24].

The infectious dose of MAP in cattle is unknown, as are shedding patterns of infected animals over time, making it difficult to interpret test results in the context of differentiating clinically affected, infected (but not necessarily infectious), infectious and non-infected cattle [24, 58].

#### The role of diagnostic tests

Diagnostic tests can play several different roles in control programmes including the early detection of infection in a herd, providing herd level assurance for risk-based trading, and for national surveillance for case-detection.

Testing permits herd-level case detection so that management recommendations can be tailored according to herd infection history. Testing to identify infected cattle for culling (test and cull) as part of a whole-herd test is an important component of on-farm control programmes. Regular screening also allows the early identification and removal of sub-clinically affected cattle, to minimize opportunities for further spread. Conversely, regular herd testing can identify low-risk herds from which to source replacement animals.

Notwithstanding the limitations of tests in identifying sub-clinically infected animals, testing may also be used for estimating herd-level and animal-level prevalence and changes in these over time. These estimates may be useful for monitoring progress in reducing the number of test-positive animals in the herd, assuming that reducing MAP herd-level prevalence (or control below a specified prevalence over time) is a major control programme objective. Monitoring of progress in control at the farm, regional or national level can be demonstrated through herd-level prevalence estimates and an increase in the age at which animals seroconvert or test positive for MAP [65]. Evidence of a reduction in within-herd prevalence may also improve farmer engagement in voluntary programmes provided farmers understand the persistent nature of MAP in a herd.

#### Best practice on-farm control

Hygienic calf rearing practices form a key component of biocontainment methods to reduce within-herd transmission. These include cleaning calving pens after each use [56], promptly removing calves from calving pens [55], collecting colostrum cleanly from low-risk dams, cleaning of udders and avoiding feeding pooled colostrum.

On-farm pasteurisation of colostrum may reduce MAP load [66, 67], but may not remove the risk of exposure to viable bacteria completely. Early removal of calves from dams is often used as a means of reducing cow-calf transmission. Windsor and Whittington did not find evidence to support the practice of removal of neonatal calves within 12 h from their dams compared to 24 h [39], whereas McAloon and others found that herds in which calves were removed within 15 min of birth were significantly less likely to be test-positive than those where calves were removed later [55]. Culling of test–positive cows and high-risk cohorts is often practiced but should not be relied on to be effective in reducing prevalence in the absence of good calf hygiene practices.

Vaccines have been developed for MAP, which may reduce the rate of clinical manifestation and sometimes shedding, but do not reliably prevent infection [24]. Concern over potential immunological cross-reactions between MAP and *Mycobacterium bovis*, which can reduce the specificity of commonly used bovine tuberculosis diagnostic tests, has limited vaccination use in some countries. In Ireland the use of a paratuberculosis vaccine is prohibited because of this risk [68].

Minimising cattle introductions and only sourcing cattle from low-risk sources is recommended to minimise spread between herds. Exposure to manure from other cattle herds through co-grazing or deliberate application of off-farm manure should be avoided or managed to reduce the risk by spreading on pastures not grazed by young stock [60]. Farmers with mixed enterprises (dairy and beef) must ensure that measures to prevent spread are applied to both their dairy and beef herds, as well as any young stock sent to other farms for rearing and subsequent return to their parent herd.

The long incubation period of MAP and the low sensitivity of the ELISA test during the early stages of infection make testing of individual animals for MAP prior to introduction problematic, with negative results providing little assurance to prospective purchasers. National or regional herd-level testing to provide assurance of freedom from infection in the herd from which cattle are to be sourced is preferable.

In summary, undertaking sound calf hygiene practices and culling of clinically-affected, test positive or other high-risk cattle can minimise within-herd transmission. Minimising cattle introductions, sourcing cattle from low-risk herds and reducing exposure to off-farm manure and colostrum are the primary methods to minimise between-herd transmission.

## Abbreviations

AHI: Animal Health Ireland
bTB: Bovine tuberculosis
DAFM: Irish Department of Agriculture, Food and the Marine
IJCP: Irish Johne’s Control Programme
IAP: International Association for Paratuberculosis
JDIG: Johne’s disease Implementation Group
MAP: *Mycobacterium avium* subspecies *paratuberculosis*
PCR: Polymerase Chain Reaction
UK: United Kingdom
USA: United States of America
VRAMP: Veterinary Risk Assessment and Management Plan
OIE: World Organisation for Animal Health
